# Predicting and optimizing control parameters of stir casting of Al alloy/MWCNT/RHA composite using artificial neural network and Taguchi-Grey relational analysis for multi-objective outcomes

**DOI:** 10.1371/journal.pone.0343970

**Published:** 2026-03-12

**Authors:** Nitin Srivastava, Manoj Kumar Yadav, Selsam Ajith Arul Daniel, Alok Bhadauria, Lavish Kumar Singh

**Affiliations:** 1 Department of Mechanical Engineering, Sharda University, Greater Noida, Uttar Pradesh, India; 2 Department of Mechanical Engineering, Ajay Kumar Garg Engineering College, Ghaziabad, Uttar Pradesh, India; 3 Department of Mechanical Engineering, Vels Institute of Science, Technology and Advanced Studies, Chennai, Tamil Nadu, India; 4 Department of Mechanical and Industrial Engineering, Manipal Institute of Technology Bengaluru, Manipal Academy of Higher Education, Manipal, Karnataka, India; 5 School of Engineering, Jawaharlal Nehru University, New Delhi, Delhi, India; Zhejiang University, CHINA

## Abstract

In the present investigation, the influence of various casting parameters viz. stirrer time, stirrer speed, and processing temperature and reinforcement content on the mechanical properties of AlP0507/CNT/RHA composite is assessed. The optimum parameter combination that produces greater multi-objective performance was obtained using the GRA method. The comparison of all R^2^-score showed that the ANN model is best fitted to predict the tensile strength of HAMMC with highest R^2^- score of 99.65%. GRA established that the MWCNT content has most significant influence on the response parameters followed by stirring time, RHA content, stirring speed and processing temperature; and the best properties of stir cast HAMMCs was obtained by the combination A2B3C3D2E2. ANNOVA performed on GRA indicated that MWCNT content with contribution of 48.26% exerted maximum impact on the properties of the fabricated HAMMC, followed by stirring time with contribution 19.4%. Processing temperature contributed least with meagre contribution of 2.17%. The predicted value of GRG (0.830775) was found very close to the GRG value of the highest-ranked experiment (0.79643454) confirming the accuracy of the optimization and its validation. The improvement in GRG value by 0.09792454 shows that the optimized parameters provided the optimal results and can be recommended.

## 1. Introduction

The importance of hybrid aluminium metal matrix composite (HAMMC) has significantly increased in modern manufacturing era. Usually, ceramic particles are added to aluminium alloys to produce HAMMCs, to alter mechanical strength, wear resistance, and corrosion characteristics as per the requirements [1–3]. HAMMCs have been widely used owing to their inimitable mechanical and physical properties. They have emerged as a new alternative to traditional ferrous materials in sectors such as autos, marine, and aerospace. Although HAMMCs provide several benefits, the process of machining them, which includes milling, turning, and drilling, presents difficulties because of the existence of tough reinforcements in the metal matrix. These challenges lead to outcomes such as heightened tool erosion, reduced surface integrity, and machine oscillations [4–6]. Therefore, the ease of machining becomes essential for effectively incorporating of HAMMCs into prospective applications.

While investigating a novel material in the stir-casting process, analyzing the effect of process factors on response variables, and forecasting the response variable, as well as optimizing process parameters become extremely important. Regression equations are often used to predict response parameters in the stir-casting process by analyzing experimental data. However, Singhal et al. [7] revealed that artificial neural network(ANN) was more successful than regression equations in anticipating the link between reinforcement content, stir casting speed, stir duration, and processing temperature during fabrication of HAMMCs. According to Varol et al. [8], ANN is a very efficient technique for predicting output variables with little error in determining the mechanical and physical properties of the prepared component. They used ANN model for predicting the response variable in the stir-casting process Al2024-B_4_C composite. In another experimental investigation carried out by Srinivas [9], it was shown that the feed rate has a considerable impact on the hardness, toughness, and tensile characteristics of Al/MWCNT composite during the stir-casting process.

A new effective approach, known as the Taguchi-grey relational analysis (GRA), is being used by the researchers to investigate, understand, and optimize the impact of processing parameters on various properties [10–13]. Kumar et al. [11] utilized GRA to examine the effect of varying weight percentages of RHA/MWCNT and various casting parameters on the mechanical characteristics of HAMMC. The MWCNT content was found to be the most significant factor affecting the mechanical characteristics and the desired mechanical properties were achieved at 10% RHA and a stirrer speed feed rate of 200 rpm. GRA can be used to optimize not only casting parameters but also welding and machining parameters. For instance, Tomadi et al. [12] used Taguchi and GRA methodologies to investigate the influence of cutting parameters on the performance characteristics of AlSi/AlN composite and it was inferred that lower surface roughness, longer tool life, lower cutting force and higher material removal could be attained by using uncoated carbide with cutting speed, feed, depth of cut and AlN content being 240 m/min, 0.4 mm/tooth, 0.3 mm and 15 vol.%, respectively. Sankar et al. [13] used GRA to conclude that among all weld parameters, the weld current exerted most significant impact while welding AISI 310 metals. Furthermore, GRA is a technique that does not need intricate calculations for multi-objective optimization, rendering it a fast and efficient approach for generating outcomes within a short time-frame. The mechanical properties of cast products can be significantly enhanced by carefully selecting the input process parameters, such as stirrer speed, stirrer duration etc. However, it is important to acknowledge that these parameters, under certain conditions, might also have negative consequences on stir casting, possibly affecting the quality of the output [14–16].

Most of the researchers have primarily concentrated on analyzing the influence of the reinforcement content on different properties, while only a small number of studies have explored the impact of various stir-casting parameters on response variables. Furthermore, a comprehensive review of the literature indicates that many scholars have investigated the stir-casting technique of different metal matrix composites and alloys, usually focusing on just one or two dependent variables. It is worth mentioning that the investigation of the stir-casting method, using five components with three levels, has very rarely been documented. To fill this research gap, this study focuses on the examination of the influence of each input parameter related to casting viz. stirrer time, stirrer speed and processing temperature and reinforcement content on three most important response variables: hardness, toughness, and tensile strength of AlP0507/CNT/RHA composite. In addition, a regression model and an ANN model was designed to predict response parameters, and their performances were evaluated and compared. Furthermore, the optimum parameter combination that produces greater multi-objective performance was obtained using the GRA method. The primary objective of this comprehensive methodology is to provide a better and integrated understanding of the stir-casting process and its parameter optimization.

## 2. Experimental details

### 2.1. Materials

AlP0507 is taken as the base metal for the proposed work, whose chemical composition is depicted in Table 1. MWCNT and RHA were selected as the reinforcement. MWCNTs were reinforced at three specific weight percentages: 1%, 2% and 3%. The weight fraction of RHA was taken as 2%, 4% and 6%.

**Table 1:** Chemical Composition of AlP0507.

Element	Si	Fe	Mn	Mg	Cu	Zn	Ti	V	Ga	Na	Cr	Zr	Ni	Pb	Sr	Ca	Al
**Weight%**	0.0408	0.0584	0.0014	0.0005	0.0005	0.0020	0.0031	0.0129	0.0109	0.0020	0.0015	0.0004	0.0032	0.001	0.0001	0.0001	99.88

### 2.2. Experimental setup and process parameters

**Fig 1:**
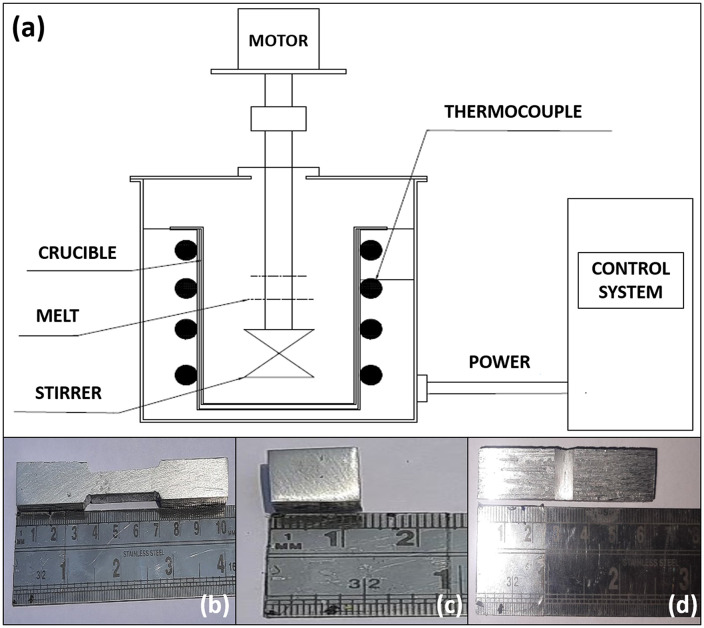
(a) Schematic diagram of the stir casting experimental setup and digital photograph of a sample cut out for (a) tensile test, (c) hardness test and (d) impact test.

The stir-casting furnace is used to produce the hybrid Al/CNT/RHA metal matrix composites. Fig 1a displays the schematic diagram of the experimental setup used for the stir-casting process, while Fig 1(b-d) depicts the digital photograph of a sample cut out for different mechanicals tests. The input process parameters, selected after numbers of experimental trials, are detailed in Table 2. These factors include the percentage of MWCNT by weight, the percentage of RHA by weight, stirrer speed, stirrer time, and the processing temperature with three levels of each parameter.

**Table 2:** Control parameters and corresponding levels used in the present study.

S. No.	Factors	Nomenclature	Unit	Values		
				I	II	III
1	wt.% of MWCNT	A	%	1	2	3
2	wt.% of RHA	B	%	2	4	6
3	Stirrer time	C	minute	5	10	15
4	Stirrer speed	D	rpm	100	200	300
5	Processing temperature	E	°C	750	800	850

## 3. Experimental design and optimization

### 3.1. Taguchi S/N ratio analysis

Taguchi L27 orthogonal array technique for experimental design was used to minimize the number of trials with combination of input parameters. To evaluate the signal-to-noise ratio (S/N) for the improvement of tensile strength, toughness, and hardness, ‘larger is better’ criteria was selected, as shown in the following equation.


$$\text{S}/\text{N Ratio}=\;-\text{10log}\left(\frac{\text{1}}{\text{n}}\sum_{\text{i}=\text{1}}^{\text{n}}\frac{\text{1}}{{\text{y}}_{\text{i}}^{\text{2}}}\right)$$ (1)

where ‘i’ represents the number of experiments and ‘y_i_’ represents the experimental result of the i^th^ experiment.

### 3.2. Artificial neural network model

**Fig 2:**
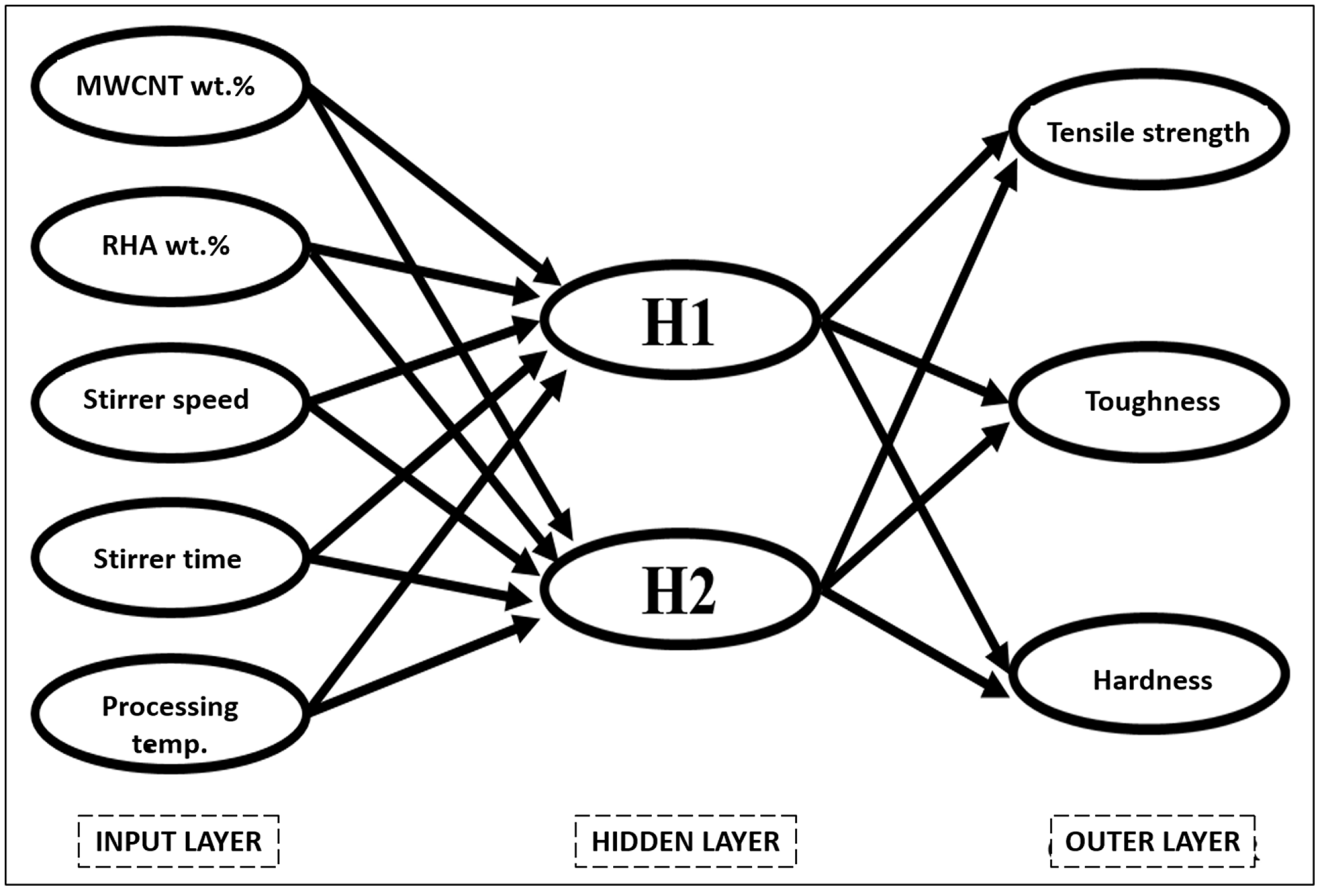
Architecture of artificial neural network.

ANN is a computer model that emulates the information-processing operations of the brain. The system consists of several interconnected processing components known as neurons, which work concurrently to tackle a particular problem. Fig 2 illustrates the arrangement of a three-layer ANN.

The input neurons correspond to the wt.% of MWCNT, wt.% of RHA, stirrer speed, stirrer time, and processing temperature and the output neurons correspond to the tensile strength, toughness and hardness. The input and output values are trained using a backpropagation approach, using 27 experimental data sets, as shown in Table 3, for both training and validation purposes.

**Table 3:** L27 orthogonal array of input parameters and corresponding mechanical properties.

S. No.	MWCNT wt.%	RHA wt.%	Stirrer Time (min)	Stirrer Speed (rpm)	Processing Temperature (°C)	Tensile Strength (MPa)	Toughness (J)	Hardness (BHN)
Input Parameters								
1	1	2	5	100	750	41.9382	49.6860	41.2892
2	1	2	5	100	800	42.0761	49.5713	41.5109
3	1	2	5	100	850	41.8684	49.7428	41.3637
4	1	4	10	200	750	42.2118	50.1301	41.6557
5	1	4	10	200	800	42.4770	50.2910	41.7981
6	1	4	10	200	850	42.3454	50.0758	41.5836
7	1	6	15	300	750	42.6067	50.1301	41.9382
8	1	6	15	300	800	42.8603	49.9662	42.0761
9	1	6	15	300	850	42.7344	50.1030	42.1442
10	2	2	15	200	750	42.2789	50.2910	41.9382
11	2	2	15	200	800	42.4770	50.2377	42.2118
12	2	2	15	200	850	42.3454	50.3703	42.4115
13	2	4	5	300	750	42.6067	50.6296	42.3454
14	2	4	5	300	800	42.4770	50.7564	42.2118
15	2	4	5	300	850	42.5421	50.5009	42.4770
16	2	6	10	100	750	42.8603	50.1030	42.4115
17	2	6	10	100	800	42.9844	50.2644	42.5421
18	2	6	10	100	850	42.7344	50.0758	42.6708
19	3	2	10	300	750	42.8603	50.3175	42.3454
20	3	2	10	300	800	42.4115	50.3966	42.5421
21	3	2	10	300	850	42.2118	50.1301	42.2118
22	3	4	15	100	750	42.0761	50.4749	42.2789
23	3	4	15	100	800	42.3454	50.3966	42.1442
24	3	4	15	100	850	42.2118	50.5526	42.0761
25	3	6	5	200	750	42.5421	50.1841	42.0074
26	3	6	5	200	800	41.9382	50.3175	42.2118
27	3	6	5	200	850	42.0761	50.0485	41.9382

### 3.3. Grey relational analysis

The combined response parameters comprehend more effectively using Taguchi design in combination with GRA. Three primary components make up the GRA analysis process. First of all, the output function was normalized. The normalization equation that was chosen takes the ‘larger is better’ approach, as shown in equation (2).


$$\;{\text{Y}}_{\text{ij}}=\frac{\left({\text{Z}}_{\text{ij}}-\text{min}\left({\text{Z}}_{\text{ij}}\right)\right)}{\text{max}\left({\text{Z}}_{\text{ij}}\right)-\text{min}\left({\text{Z}}_{\text{ij}}\right)}$$ (2)

The term “Z_ij_” refers to the value that was computed based on the experimental data, whereas, the term “min (Z_ij_)” represents the value that received the least amount of information from the experiment.

Along the same lines, max (Z_ij_) represents the highest possible value that may be derived from the L27 Taguchi design data. A calculation is made using the equation (3) to determine the normalized data.


$$\;{\text{GRC}}_{\text{ij}}=\frac{\left(\delta_{\text{min}}-\gamma \delta_{\text{max}}\right)}{\left(\delta_{\text{ij}}-\gamma \delta_{\text{max}}\right)}$$ (3)

Regarding the stir-casting process, a higher Grey Relational Grade (GRG) implies that an appropriate blend of good mechanical characteristics has been achieved. The grade determined by the GRG is calculated by utilizing the following equation.


$${\text{GRG}}_{\text{i}}{=}_{\text{n}}^{\text{1}}\epsilon {\text{GRC}}_{\text{ij}}$$ (4)

## 4. Results and discussion

### 4.1. Microstructural Investigation

**Fig 3:**
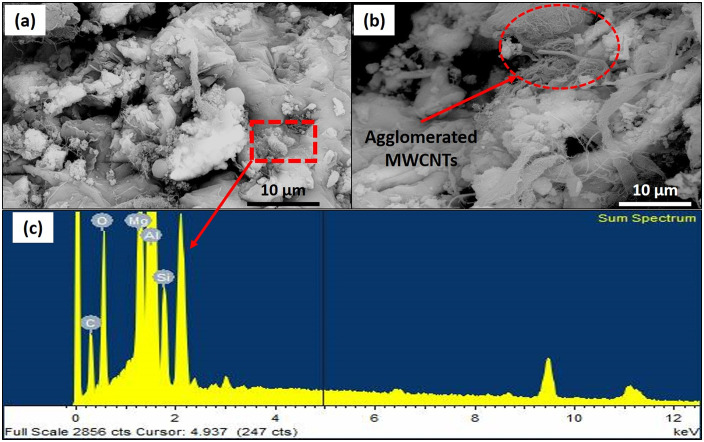
SEM images of (a) Al/2 wt.% RHA/2 wt.% MWCNT composite, and (b) Al/2 wt.% RHA/3 wt.% MWCNT composite and (c) EDS graph of the portion marked in (a).

Scanning electron microscopy (SEM) was performed over the fabricated HAMMCs to assess the morphology and dispersion of the reinforcements. The SEM images of the fabricated composites along with the Energy dispersive spectroscope (EDS) results are shown in Fig 3.

The judicious utilization of ultrasonication and other precautionary measures led to a uniform dispersion of MWCNTs in the prepared composites containing up to 2 wt.% CNT (Fig 3a). However, significant agglomerations of CNTs were observed in the composites were the nano-reinforcement content was 3 wt.% (Fig 3b). The effect of CNT dispersion and agglomeration on the mechanical properties of the composite will be discussed in the subsequent sections. The EDS peaks, shown in Fig 3c, confirms the presence of RHA in the hybrid composite.

### 4.2. Taguchi model analysis

**Fig 4:**
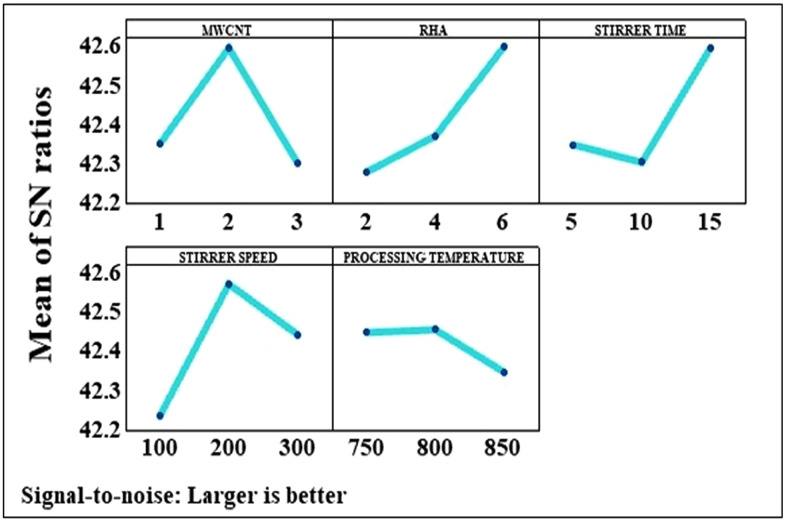
Variation in the mean S/N ratio for tensile strength.

Optimal tensile strength was achieved corresponding to the parameter configuration, represented by the parameter combination A_2_B_3_C_3_D_2_E_2_. Therefore, optimal tensile strength was achieved at a concentration of 2 wt.% MWCNT, 6 wt.% RHA, stirring time of 15 min, stirring speed of 200 rpm, and processing temperature of 800 °C. Fig 4 depicts the variation in the S/N ratio for tensile strength as a result of MWCNT addition in the AMMC. The presence of sp2 bonds between the individual carbon atoms of MWCNT makes it a promising strengthening material [17]. Load transfer mechanism also plays a decisive role for increasing the tensile strength of HAMMC [18]. Therefore, adding 1–2 wt.% MWCNT improved the tensile strength of the HAMMC. However, there exists a substantial difference in the surface energy of AlP0507 and MWCNT, which results in poor wettability and poses challenges when adding higher proportions of CNTs. When the amount of nanoparticles in the matrix exceeds a threshold limit, it leads to agglomeration. The agglomerations result in porosity which acts as a site of stress concentration, thereby, affecting load transfer and overall strength. In this study, adding 3 wt.% of MWCNT led to agglomeration in the AlP0507 matrix, which consequently resulted in a reduction in the tensile strength, as depicted in Fig 4.

The RHA has high silica content which acts as mechanical property enhancer. Along with this, RHA is a light weight reinforcement and has high melting point and low coefficient of expansion, which makes it a promising reinforcement material [19]. RHA also acts as a barrier for the advancement of dislocation in the metal matrix, when the load is applied. This is primarily attributable to the enhanced bonding at the AlP0507-RHA interface [20,21]. As a result, adding RHA led to a monotonous improvement in the tensile strength of HAMMC, as also illustrated in Fig 4.

Achieving homogeneous distribution of particles in the melt is a major problem frequently faced in stir casting process, which is controlled by stirring parameters. A higher stirring time of 15 min was found to produce the best results for tensile strength. However, when the stirring speed was increased from 200 to 300 rpm, the S/N ratio for tensile strength dips.

The decrease can be explained by the slurry’s increased agitation severity, which leads the to the clustering of MWCNTs and encourages gas entrapment in the slurry- a process that results in high porosity and blow holes. Moses et al. [15] have also reported that high stirring speed creates a strong vortex, which results in turbulence and suction of air bubbles. The stirring action should be slow to prevent the formation of vortex at the surface of the melt, and care must be taken not to break the surface too often, which could contaminate the bath with dross.

It has been reported that a judicious combination of stirring speed and stirring time is required to achieve homogenous dispersion of reinforcement particles. For instance, in their study, Prabu et al. [22] examined the impact of stirring duration and speed on the hardness of an aluminum matrix composite reinforced with silicon carbide. They chose to stir for 5, 10 and 15 min at a speed of 500, 600, and 700 rpm. The experimental investigation found that the ideal stirring speed and duration for achieving a consistent hardness value throughout the composite is 600 rpm and 10 min, which validates the uniform dispersion of SiC particles throughout the aluminum matrix.

An increase in processing temperature from 800 to 850 °C resulted in decrease of tensile strength. The occurrence might be related to the considerable formation of Built-Up-Edge (BUE) at high weight percentages of RHA, which ultimately leads to an increase in tensile strength [23].

**Fig 5:**
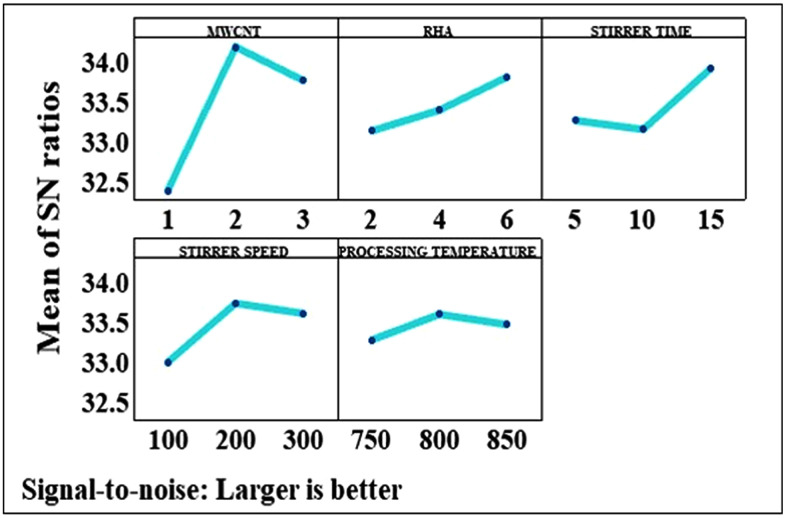
Variation in the mean S/N ratio for hardness.

**Fig 6:**
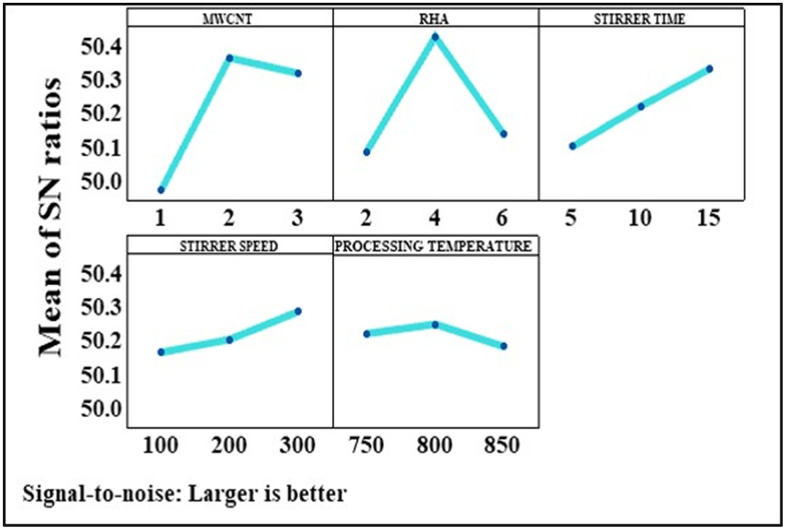
Variation in the mean S/N ratio for toughness.

However, higher processing temperatures result in the disappearance of the BUE, which in turn results in a decrease in the tensile strength. A similar combination of optimal input parameters, i.e., A_2_B_3_C_3_D_2_E_2_, was identified for achieving enhanced hardness, which is evident from Fig 5. Fig 6 illustrates the optimal set of input parameters that provides the best fit result for the toughness of the HAMMC and that set of parameters is represented by A_2_B_2_C_3_D_3_E_2_. It was observed that the toughness got increased upon increasing the CNT content from 1 wt.% to 2 wt.%. The addition of MWCNT inhibits crack propagation by spreading along the grain boundaries of aluminium metal matrix. But due to poor wettability and gravity segregation, as discussed earlier, further addition of MWCNT led to the degradation of toughness. There are two major differences between the optimal parameters found for toughness and tensile strength or hardness. First, although increasing the RHA content from 2 to 4 wt.% resulted in enhanced toughness, but further increasing the content to 6 wt.% exerted a detrimental impact on the toughness. Second, the S/N ratio in case of both the stirring parameters, stirring time and speed, varied in an approximately linear manner.

### 4.3. Regression model analysis

Regression model is used to determine the relationship between the unknown parameters, independent variables, and dependent variables. The regression models that were derived from the generic linear model are represented by equations (5) to (7), whose variables are described as: ‘A’ stands for the weight percentage of MWCNT, ‘B’ stands for the weight percentage of RHA, ‘C’ stands for the stirring time in minutes, ‘D’ stands for the stirrer speed in rpm, and ‘E’ stands for the processing temperature in °C. In the subsequent sections, similarities and differences between the expecte7700d experimental results obtained via the regression equations and the results provided by the ANN model will be assessed and analysed.


TENSILE  STRENGTH = 133.6 − 0.389 × A + 1.222 × B + 0.367 × C + 0.01556 × D − 0.0156 × E (5)

TOUGHNESS = 308.7 + 6.39 × A + 0.42 × B + 0.844 × C + 0.0211 × D − 0.0144 × E (6)

HARDNESS = 102.0 + 3.500 × A + 0.833 × B + 0.311 × C + 0.01444 × D + 0.0111 × E  (7)

### 4.4. Artificial neural network analysis

**Fig 7:**
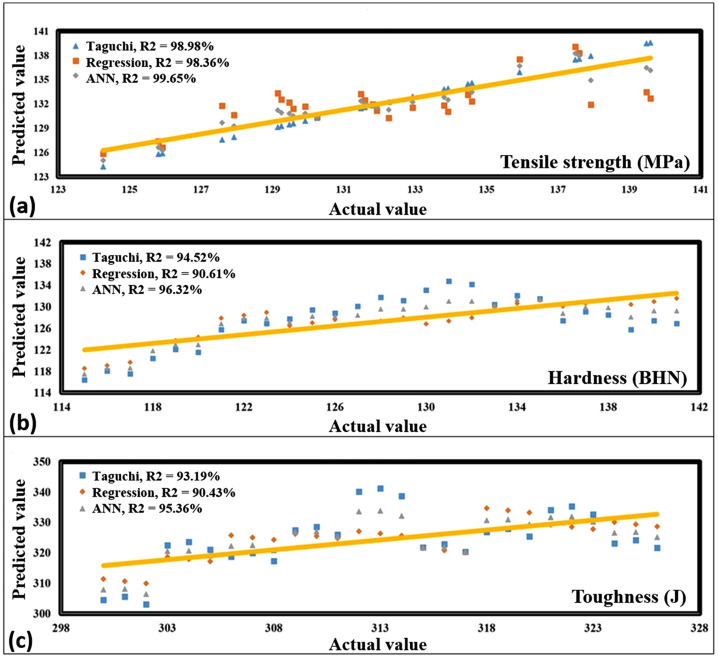
Comparison of the projected values with the values obtained via experimentation, regression, and ANN model for (a) tensile strength, (b) hardness and (c) toughness.

ANN is an extremely efficient computing approach that can be used to create optimal solutions in a variety of conditions. It is a component of connectionism systems. The effectiveness of these systems depends on specialized training, during which the system learns from data to perform its functions effectively. During this investigation, the ANN model was trained and the predicted response values obtained from the ANN were compared with the outcomes of the mathematical regression model and the Taguchi method. Fig 7 provides a visual depiction of the findings of this comparative investigation. The coefficient of determination, R^2^-score, is used to evaluate the prediction accuracy of each model. Higher the R^2^-score, better is the performance of the model [24,25].

When it comes to the prediction of tensile strength, the comparison plot in Fig 7 reveals that the R^2^- score for the ANN is 99.65%, while the score for the Regression and Taguchi models is 98.36% and 98.98%, respectively. The comparison of all R^2^-score shows that the ANN model, which captures the non-linear relationship between input and output, is best fitted to predict the tensile strength of HAMMC. Similar results were obtained for hardness as well as toughness, where highest R^2^-score was found in case of ANN model followed by Taguchi and Regression model, respectively. The findings unanimously suggest that the ANN model is the most effective of all the considered models in forecasting various mechanical properties such as tensile strength, hardness, and toughness.

#### 4.4.1. Cross-validation.

**Fig 8:**
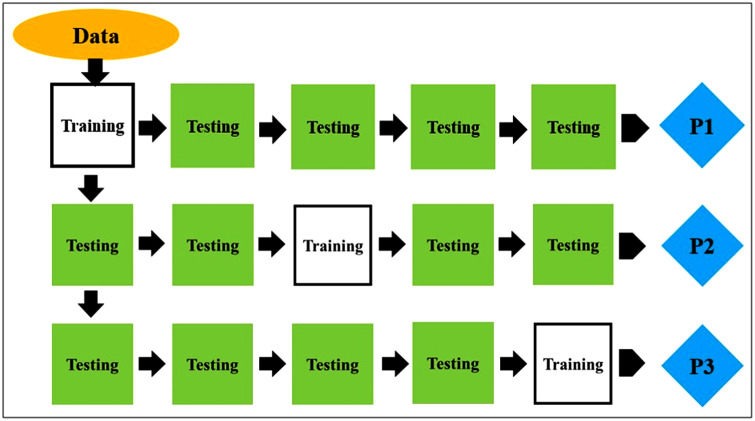
Schematic diagram depicting 3 × 5-fold cross-validation strategy.

A 3 × 5-fold cross-validation strategy, which is also commonly known as the rotation estimation, has been used to assess the degree of accuracy of the model’s prediction. Schematic diagram depicting this cross-validation strategy is presented in Fig 8. During the learning process, it is very important to evaluate the degree to which a model can successfully adapt to a variety of inputs. During the first stages of cross-validation, the dataset is segmented into a large number of subgroups, with each subset containing an equal number of samples throughout the process. One of the subsets, which is referred to as the training set, is used to generate the data that is analysed, while the other subset is kept aside for validation purposes. One of the distinguishing features of this method is its iterative nature, in which many subsets take turns serving as the validation set to reduce the amount of unpredictability. Table 4 displays the results of the calculations and verifications by considering the means of all the subgroups.

**Table 4:** Outcome of the cross-validation strategy; average of the subsets.

Response Parameters	Average (%)
Tensile Strength	98.98
Toughness	93.19
Hardness	99.52

The mean prediction accuracy is the average of all the prediction values of subsets, which is tabulated in Table 4. It is possible to verify the expected outcome of the training and testing model by computing the mean of each response variable. The average of each response variables validates the predicted outcome of the training model. This methodological approach is quite comprehensive, which makes it easier to comprehend the accuracy of the model as well as its capacity to generalize across all the experimental replications.

### 4.5. Grey relational analysis

**Fig 9:**
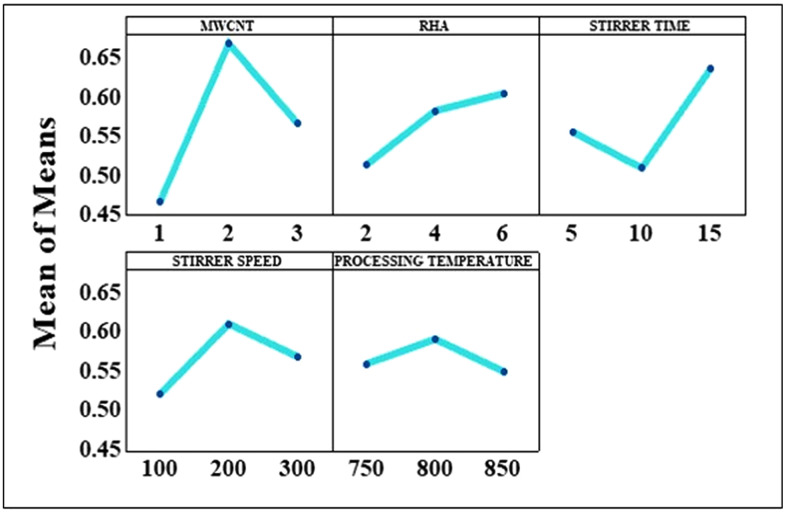
Variation in the mean S/N ratio for grey relational grade.

It is well-known that the Taguchi approach is a robust statistical strategy that is capable of discovering and resolving challenges that are associated with single-objective optimization. On the other hand, the GRA is used while attempting to identify the combination of response functions that yields the best results. The standardized values for the output variables are shown in Table A1, which enables a comprehensive analysis of the optimization process to be carried out. The GRG is shown in Table A2, which is derived by taking the average of the sum of the grey relational coefficient (GRC) variables. When the GRG values are on the higher side, it indicates that the process components have been combined most advantageously. Detailed response table and variation in the mean S/N ratio for grey relational grade is shown in Table 5 and Fig 9 respectively.

**Table 5:** Response table for grey relational grade.

Level	MWCNT	RHA	Stirrer time	Stirrer speed	Processing temperature
1	0.4598	0.5057	0.5478	0.5132	0.5507
2	0.6582	0.5730	0.5008	0.6022	0.5817
3	0.5560	0.5953	0.6254	0.5586	0.5416
Delta	0.1984	0.0896	0.1246	0.0890	0.0401
Rank	1	3	2	4	5

As demonstrated by the grey relational grade response table, the mean effect graph also illustrates that the combination of response parameters is more advantageous as compared to the individual parameters. It is also clear that the MWCNT content has the most significant influence on the response parameters followed by stirring time, RHA content, stirring speed and processing temperature. A_2_B_3_C_3_D_2_E_2_ is the optimal representation of the combination of input parameters for obtaining best properties of stir cast HAMMCs, as concluded by the GRA.

### 4.6. ANOVA for grey relational grade

ANOVA is used to determine the significance of process parameters and the proportional influence they have on the quality and characteristics of the product. Presented in Table 6 are the results of the ANOVA performed on GRA. MWCNT content with contribution of 48.26% exerted maximum impact on the properties of the fabricated HAMMC, followed by stirring time with contribution 19.4%. Processing temperature contributed least with meagre contribution of 2.17%. The P-values also suggests that the effect of MWCNT content, stirrer time and RHA content is significant.

**Table 6:** The ANOVA results of the analysis for grey relational grade.

Source	DF	Seq SS	Contribution	Adj SS	Adj MS	F-Value	P-Value
MWCNT (wt.%)	2	0.177211	48.26%	0.177211	0.088606	39.4	0
RHA (wt.%)	2	0.039176	10.67%	0.039176	0.019588	8.71	0.003
Stirrer Time (min)	2	0.071227	19.40%	0.071227	0.035614	15.84	0
Stirrer Speed (rpm)	2	0.035667	9.71%	0.035667	0.017834	7.93	0.004
Processing Temperature (°C)	2	0.00796	2.17%	0.00796	0.00398	1.77	0.202
Error	16	0.035982	9.80%	0.035982	0.002249		
Total	26	0.367224	100.00%				

### 4.7. Confirmation experiment

To enhance stir-casting quality, the GRA technique is used to determine the procedure parameters that provide the best possible combination of results. In the last stage of the GRA process, confirmation studies are used to validate the ideal condition that has been obtained for a variety of quality criteria. These confirmation trials may be represented by the equation [26], which can be expressed as following:


$$\rho =\;\rho_{\text{t}}+\sum_{\text{i}=\text{0}}^{\text{n}}\left(\rho_{\text{m}}-\rho_{\text{t}}\right)$$ (8)

In the equation that has been supplied, the symbol ρ_t_ represents the overall average of the GRG, ρ_m_ stands for the average of the GRG at the ideal level, and ‘n’ indicates the number of stir-casting parameters. It is found that the combination of A_2_B_3_C_3_D_2_E_2_ is the optimal combination for the input control parameters. The grey relational grade was obtained by using equation (8).

There is a complete review of the confirmation tests that were carried out for the response parameters which is tabulated in Table 7. According to the findings acquired from GRA, the experimental values of MWCNT weight percent, RHA weight percent, stirrer time, stirrer speed, and processing temperature have been significantly improved. This is quite evident from the table, which displays the results. The predicted value obtained from equation (8) is 0.830775 which is very close to the value of the highest-ranked experiment number 17, i.e., 0.79643454. So, on this basis, it can be stated that the result of the optimization is validated. The improvement in the GRG value by 0.09792454 indicates that the optimized parameters provided the optimal results and can be recommended. According to GRA and GRG data, a considerable increase in tensile strength by 9.75%, toughness by 7.78% and hardness by 34.31% was observed. As a result, GRA and GRG were found to be helpful methods in multi-objective optimization issues. The results of the confirmatory studies are quite encouraging, and response quality has improved.

**Table 7:** Comparison between initial and optimal stir-casting parameters.

	Initial stir-casting Parameters	Optimal stir-casting Parameters	
		Prediction	Experiment
Level	A_2_B_3_C_2_D_1_E_3_	A_2_B_3_C_3_D_2_E_2_	A_2_B_3_C_3_D_2_E_2_
Tensile Strength	39.16536		42.9844
Toughness	46.63487		50.2644
Hardness	31.67354		42.5421
GRG	0.69851	0.830775	0.79643454

## 5. Conclusions

In the present investigation, the influence of various casting parameters viz. stirrer time, stirrer speed, and processing temperature and reinforcement content on the mechanical properties of AlP0507/CNT/RHA composite was assessed and analysed. In addition, a regression model and an ANN model was designed to predict response parameters, and their performances were evaluated and compared. Furthermore, the optimum parameter combination that produces greater multi-objective performance was obtained using the GRA method. The findings of the study are summarized in the following observations and conclusions.

- The judicious utilization of ultrasonication and other precautionary measures led to a uniform dispersion of MWCNTs in the prepared composites containing up to 2 wt.% CNT. However, significant agglomerations of CNTs were observed in the composites containing 3 wt.% nano-reinforcement.
- Taguchi model analysis revealed that the optimal tensile strength and hardness was achieved corresponding to the parameter configuration, A_2_B_3_C_3_D_2_E_2_. However, optimal set of input parameters that provided the best fit result for the toughness of the prepared HAMMCs was represented by A_2_B_2_C_3_D_3_E_2_.
- The R^2^- score for the ANN was calculated to be 99.65%, while the score for the Regression and Taguchi models was 98.36% and 98.98%, respectively. The comparison of all R^2^-score showed that the ANN model, which captures the non-linear relationship between input and output, is best fitted to predict the tensile strength of HAMMC.
- GRA established that the MWCNT content has most significant influence on the response parameters followed by stirring time, RHA content, stirring speed and processing temperature; and the best properties of stir cast HAMMCs was obtained by the combination A_2_B_3_C_3_D_2_E_2_.
- ANNOVA performed on GRA indicated that MWCNT content with contribution of 48.26% exerted maximum impact on the properties of the fabricated HAMMC, followed by stirring time with contribution 19.4%. Processing temperature contributed least with meagre contribution of 2.17%.
- The predicted value of GRG (0.830775) was found very close to the GRG value of the highest-ranked experiment (0.79643454) confirming the accuracy of the optimization and its validation. The improvement in GRG value by 0.09792454 shows that the optimized parameters provided the optimal results and can be recommended.

## Supporting information

S1 File **Table A.1.** Normalized value for response parameters. **Table A.2.** Grey Relational Coefficients and Grades.
(ZIP)
