# Supplementary material for: Predicting and optimizing control parameters of stir casting of Al alloy/MWCNT/RHA composite using artificial neural network and Taguchi-Grey relational analysis for multi-objective outcomes
Source: PLoS One. 2026 Mar 12;21(3):e0343970. doi: 10.1371/journal.pone.0343970 (PMC12981493; doi:10.1371/journal.pone.0343970)
Supplement: S1 File — Table A.1. Normalized value for response parameters. Table A.2. Grey Relational Coefficients and Grades. (ZIP) [file pone.0343970.s001.zip › Supporting Information/Table A.1. Supporting information.docx]

**Table A.1.** Normalized value for response parameters

| **S. No.** | **Tensile Strength** | **Toughness** | **Hardness** |
| --- | --- | --- | --- |
| 1 | 0.062544803 | 0.09676105 | 0 |
| 2 | 0.186111111 | 0 | 0.160521491 |
| 3 | 0 | 0.144666731 | 0.053963852 |
| 4 | 0.307706093 | 0.471515799 | 0.265303087 |
| 5 | 0.545340502 | 0.607251958 | 0.368366874 |
| 6 | 0.427419355 | 0.425706006 | 0.213130587 |
| 7 | 0.66155914 | 0.471515799 | 0.469768269 |
| 8 | 0.888799283 | 0.33321843 | 0.56956005 |
| 9 | 0.775985663 | 0.448646692 | 0.618868147 |
| 10 | 0.367831541 | 0.607251958 | 0.469768269 |
| 11 | 0.545340502 | 0.562285295 | 0.66779252 |
| 12 | 0.427419355 | 0.67418889 | 0.812321878 |
| 13 | 0.66155914 | 0.89299747 | 0.764513661 |
| 14 | 0.545340502 | 1 | 0.66779252 |
| 15 | 0.603673835 | 0.784409661 | 0.859769275 |
| 16 | 0.888799283 | 0.448646692 | 0.812321878 |
| 17 | 1 | 0.58480311 | 0.906861257 |
| 18 | 0.775985663 | 0.425706006 | 1 |
| 19 | 0.888799283 | 0.62963226 | 0.764513661 |
| 20 | 0.486648746 | 0.696366042 | 0.906861257 |
| 21 | 0.307706093 | 0.471515799 | 0.66779252 |
| 22 | 0.186111111 | 0.762497707 | 0.716339095 |
| 23 | 0.427419355 | 0.696366042 | 0.618868147 |
| 24 | 0.307706093 | 0.828038022 | 0.56956005 |
| 25 | 0.603673835 | 0.517041058 | 0.519862161 |
| 26 | 0.062544803 | 0.62963226 | 0.66779252 |
| 27 | 0.186111111 | 0.402693293 | 0.469768269 |
