# Supplementary material for: Predicting and optimizing control parameters of stir casting of Al alloy/MWCNT/RHA composite using artificial neural network and Taguchi-Grey relational analysis for multi-objective outcomes
Source: PLoS One. 2026 Mar 12;21(3):e0343970. doi: 10.1371/journal.pone.0343970 (PMC12981493; doi:10.1371/journal.pone.0343970)
Supplement: S1 File — Table A.1. Normalized value for response parameters. Table A.2. Grey Relational Coefficients and Grades. (ZIP) [file pone.0343970.s001.zip › Supporting Information/Table A.2. Supporting information.docx]

**Table A.2.** Grey Relational Coefficients and Grades

| **S. No.** | **Tensile Strength** | **Toughness** | **Hardness** | **GRG** | **Rank** |
| --- | --- | --- | --- | --- | --- |
| 1 | 0.347836928 | 0.356318502 | 0.333333333 | 0.345829588 | 27 |
| 2 | 0.380549683 | 0.333333333 | 0.373279599 | 0.362387538 | 25 |
| 3 | 0.333333333 | 0.368912954 | 0.345772822 | 0.349339703 | 26 |
| 4 | 0.419359687 | 0.486152339 | 0.404957682 | 0.436823236 | 24 |
| 5 | 0.52374695 | 0.560068437 | 0.44183931 | 0.508551565 | 18 |
| 6 | 0.466165414 | 0.465421945 | 0.388539812 | 0.44004239 | 23 |
| 7 | 0.596344982 | 0.486152339 | 0.485327703 | 0.522608342 | 17 |
| 8 | 0.818061868 | 0.428529223 | 0.537380193 | 0.594657095 | 10 |
| 9 | 0.690594059 | 0.475577521 | 0.567451963 | 0.577874515 | 11 |
| 10 | 0.441630392 | 0.560068437 | 0.485327703 | 0.495675511 | 21 |
| 11 | 0.52374695 | 0.533211218 | 0.600811711 | 0.55258996 | 14 |
| 12 | 0.466165414 | 0.605465335 | 0.72708435 | 0.599571699 | 9 |
| 13 | 0.596344982 | 0.823719796 | 0.679822281 | 0.699962353 | 4 |
| 14 | 0.52374695 | 1 | 0.600811711 | 0.70818622 | 3 |
| 15 | 0.55783265 | 0.698723798 | 0.780968454 | 0.679174968 | 6 |
| 16 | 0.818061868 | 0.475577521 | 0.72708435 | 0.673574579 | 7 |
| 17 | 1 | 0.546330528 | 0.842973091 | 0.79643454 | 1 |
| 18 | 0.690594059 | 0.465421945 | 1 | 0.718672001 | 2 |
| 19 | 0.818061868 | 0.574469821 | 0.679822281 | 0.690784656 | 5 |
| 20 | 0.493412326 | 0.622173808 | 0.842973091 | 0.652853075 | 8 |
| 21 | 0.419359687 | 0.486152339 | 0.600811711 | 0.502107913 | 20 |
| 22 | 0.380549683 | 0.677963994 | 0.638031062 | 0.565514913 | 13 |
| 23 | 0.466165414 | 0.622173808 | 0.567451963 | 0.551930395 | 15 |
| 24 | 0.419359687 | 0.74408972 | 0.537380193 | 0.5669432 | 12 |
| 25 | 0.55783265 | 0.508668245 | 0.51013233 | 0.525544409 | 16 |
| 26 | 0.347836928 | 0.574469821 | 0.600811711 | 0.507706153 | 19 |
| 27 | 0.380549683 | 0.455661117 | 0.485327703 | 0.440512834 | 22 |
